# “Mi Casa, Tu Casa”: the coati nest as a hub of *Trypanosoma cruzi* transmission in the southern Pantanal biome revealed by molecular blood meal source identification in triatomines

**DOI:** 10.1186/s13071-022-05616-w

**Published:** 2023-01-23

**Authors:** Thaíla Santos Pessanha, Heitor Miraglia Herrera, Ana Maria Jansen, Alena Mayo Iñiguez

**Affiliations:** 1 Laboratório de Biologia em Tripanosomatídeos, Instituto Oswaldo Cruz, Fundação Oswaldo Cruz, Rio de Janeiro, Rio de Janeiro Brasil; 2 Laboratório de Biologia Parasitária, Universidade Católica Dom Bosco, Campo Grande, Mato Grosso Do Sul Brasil

**Keywords:** *Tamandua tetradactyla*, *Triatoma sordida*, Molecular cloning, DTUs, Mixed infection, Wild mammals

## Abstract

**Background:**

The study of the ecology of *Trypanosoma cruzi* is challenging due to its extreme adaptive plasticity, resulting in the parasitism of hundreds of mammal species and dozens of triatomine species. The genetic analysis of blood meal sources (BMS) from the triatomine vector is an accurate and practical approach for gathering information on which wild mammal species participate in a local transmission network. South American coatis, *Nasua nasua*, act as important reservoir host species of *T. cruzi* in the Pantanal biome because of their high rate of infection and elevated parasitemia, with the main discrete typing unit (DTU) lineages (TcI and TcII). Moreover, the carnivore coati is the only mammal species to build high arboreal nests for breeding and resting that can be shared by various vertebrate and invertebrate species. Herein, we applied the sensitive and specific methodology of DNA barcoding and molecular cloning to study triatomines found in a coati nest to access the diversity of mammal species that explore this structure, and therefore, may be involved in the parasite transmission network.

**Methods:**

Twenty-three *Triatoma sordida* were collected in one coati’s nest in the subregion of Nhecolândia, Pantanal. The DNA isolated from the gut of insects was subjected to BMS detection by PCR using universal primers that flank variable regions of the cytochrome b (*cyt*b) and 12S rDNA mitochondrial genes from vertebrates. The *Trypanosoma* spp. diagnosis and DTU genotyping were based on an 18S rDNA molecular marker and also using new *cyt*b gene primers designed in this study. Phylogenetic analyses and chord diagrams were constructed to visualize BMS haplotypes, DTU lineages detected on vectors, and their interconnections.

**Results:**

Twenty of 23 triatomines analyzed were PCR-positive (86.95%) showing lineages *T. cruzi* DTU TcI (*n* = 2), TcII (*n* = 6), and a predominance of TcI/TcII (*n* = 12) mixed infection. Intra-DTU diversity was observed mainly from different TcI haplotypes. Genetic analyses revealed that the southern anteater, *Tamandua tetradactyla*, was the unique species detected as the BMS of triatomines collected from the coati’s nest. At least three different individuals of *T. tetradactyla* served as BMS of 21/23 bugs studied, as indicated by the *cyt*b and 12S rDNA haplotypes identified.

**Conclusions:**

The identification of multiple BMS, and importantly, different individuals of the same species, was achieved by the methodology applied. The study demonstrated that the southern anteaters can occupy the South American coati’s nest, serving as the BMS of *T. sordida* specimens. Since anteaters have an individualist nonsocial behavior, the three individuals detected as BMS stayed at the coati’s nest at different times, which added a temporal character to BMS detection. The TcI and TcII infection, and significantly, a predominance of TcI/TcII mixed infection profile with different TcI and TcII haplotypes was observed, due to the discriminatory capacity of the methodology applied. *Tamandua tetradactyla*, a host which has been little studied, may have an important role in the *T. cruzi* transmission in that Pantanal subregion. The data from the present study indicate the sharing of coatis’ nests by other mammal species, expanding the possibilities for *T. cruzi* transmission in the canopy strata. We propose that coatis’ nests can act as the true hubs of the *T. cruzi* transmission web in Pantanal, instead of the coatis themselves, as previously suggested.

**Graphical Abstract:**

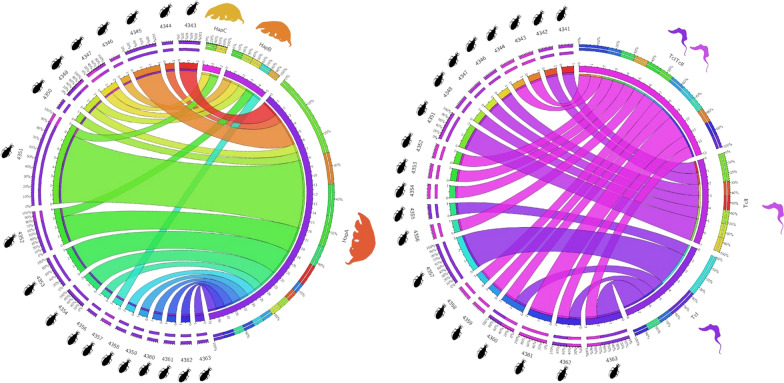

**Supplementary Information:**

The online version contains supplementary material available at 10.1186/s13071-022-05616-w.

## Background

*Trypanosoma cruzi* (Chagas, 1909) (Kinetoplastida, Trypanosomatidae) is a hemoflagellate parasite causative of American trypanosomiasis in animals and Chagas disease (DC) in humans [1]. The parasite infection occurs by two main transmission mechanisms: the vectorial-oral route, which occurs through predation of infected bugs or wild mammals, or by ingestion of contaminated food by triatomine feces; and the contaminative vectorial route, which occurs through contact with contaminated feces voided by the bugs during feeding [2]. The *T. cruzi* transmission in the wild is a complex, multi-factorial, dynamic, and non-linear network [3–5], involving hundreds of mammal species, dozens of triatomine species, and seven different lineages of the parasite [6–8].

In this complex enzootic scenario, South American coatis, *Nasua nasua* (Linnaeus, 1766) (Carnivora, Procyonidae), have been reported as a relevant host for *T. cruzi* transmission in the Pantanal biome [4, 8, 9]. South American coatis, which in Pantanal wetlands present high population densities, are scansorial mammals that feed on fruits, arthropods, small vertebrates, and even snakes [10, 11]. They are a unique mammal species that build arboreal nests for breeding and resting, which are shared by various vertebrates and invertebrates [9, 12, 13]. Coatis’ nests were found highly infested by triatomines, especially by the genera *Rhodnius* Stål, 1859, and *Triatoma* Laporte, 1832 (Hemiptera, Reduviidae), involving different developmental instars [9, 12], indicating colonization success and that South American coatis can be infected by *T. cruzi* in early life [4]. Coatis’ nests were considered a true biocenosis since they may be used concomitantly by other living beings [3, 14]. South American coatis are one of the main reservoirs of *T. cruzi* in the Pantanal wetland due to the high rate of infection, mainly by TcI and TcII discrete typing unit (DTU) lineages, in both single and mixed infections [5, 15]. In addition, because of the high parasitemia by both polar DTUs TcI and TcII, and also infection by TcIII/TcIV [3], coatis were proposed as a trypanosomatid bioaccumulator, and the key species for *T. cruzi* dispersion at the Pantanal biome [15–17].

Blood meal source (BMS) detection of hematophagous insects using DNA barcoding has been relatively recently applied to identify the species that potentially participate in the transmission cycle of the parasite. Indeed, molecular approaches have higher specificity and sensitivity than immunological assays, such as the precipitin test. In Brazil, different profiles of *T. cruzi* enzootic diseases, with diverse species involved in the transmission, were revealed in the Caatinga biome using this tool [18–21]. Two rodent species with differential roles in *T. cruzi* transmission, *Kerodon rupestris* (Wied-Neuwied, 1820) (Rodentia, Caviidae) in the wild, and *Galea spixii* (Wagler, 1831) (Rodentia, Caviidae) in linked sylvatic and domestic cycles, were the main BMS of *Triatoma brasiliensis* Neiva, 1911 complex in localities of the Rio Grande do Norte state [18]. A diversity of 20 vertebrate species were shown as BMS of *T. brasiliensis* in the Ceará state, with the highest frequency of rodents in all ecotopes [19]. In contrast, birds were the most frequent molecular BMS identified in triatomines from Bahia state, and in addition, blood of domestic dogs was highly detected in *Triatoma sordida* (Stål, 1859), the most *T. cruzi*-infected triatomine species found [20]. These three studies showed that molecular techniques can reveal the variety of BMS profiles that reflect micro-regional differences, which ultimately must be considered in epidemiological surveillance and control strategies.

Molecular techniques such as polymerase chain reaction (PCR)-heteroduplex assay [21], molecular cloning [22], and recently, next-generation sequencing (NGS) [23] have been successfully applied in BMS identification. In particular, multiple BMS not only of diverse vertebrate species but also individuals of the same species have been revealed in a few studies based on molecular cloning, [22, 24–26]. This approach could be especially relevant in the identification of mammal species that circulate among different habitats, including *T. cruzi* hosts with high infective competence, such as South American coatis, and in the coatis’ nests, a biocenosis where invertebrates and vertebrates find food and shelter, being a potentially extraordinary structure not yet explored.

Considering the complexity of the enzootic scenario in the Pantanal biome, we hypothesize that coatis’ nests functioned as important hubs of the ecological *T. cruzi* transmission. In order to elucidate the complexity of this biocenosis, we conducted BMS and *T. cruzi* DTU identification from triatomines collected in a South American coatis’ nest from the Pantanal biome, using PCR-sequencing, DNA barcoding and molecular cloning approaches.

## Methods

### Study area

Triatomines (*n* = 23) were collected from a nest of South American coatis *N. nasua* in Nhumirim Ranch (56°39′W, 18°59′S), situated in the subregion of Nhecolândia, Pantanal biome, municipality of Corumbá, Mato Grosso do Sul State, Brazil. The coati arboreal nest was identified and triatomines were collected by the specialists and coauthors of this study (AMJ and HMH). Coati nests are characterized by having open and semispherical shapes (“bird-like” nests), composed of leaves and branches from the tree in which they are located, and in the Nhumirim Ranch, the lowest nest found was 5 m above the ground, in high canopy density [9, 12, 13]. The capture and identification of triatomine bugs are described elsewhere [9]. This region is characterized by a mosaic of semi-deciduous forests, arboreal savannas, and seasonally flooded fields covered by grasslands [27]. The Pantanal is a neotropical floodplain and is known for its wide biodiversity, with two well-defined seasons: a rainy summer (October to March) and a dry winter (April to September) [28]. It is worth mentioning that this area is subjected to multi-annual cycles of high flood and severe drought years, and these seasonal flood-drought cycles are the most striking ecological phenomena of the Pantanal, resulting in drastic changes in the landscape [29].

### Triatomine processing and parasitological analysis

Triatomine nymphs of the fifth-instar stage and adults, male and female, were stored live in Falcon^®^ tubes. The specimens were characterized as *T. sordida* by a specialist from the Laboratório Nacional e Internacional de Referência em Taxonomia de Triatomíneos (LNIRT–IOC/FIOCRUZ), Rio de Janeiro, Brazil.

The abdomen of the specimens was removed with a scalpel blade and kept in sterile phosphate-buffered saline (PBS) at −20 °C, according to the LABTRIP IOC/FIOCRUZ protocol. Parasitological analysis for *Trypanosoma* spp. infection was performed through fresh examination of the gut content macerated in 0.85% saline solution supplemented with 10% antibiotic solution. The samples were examined under a light microscope for the presence of flagellated forms.

### Molecular characterization of BMS and *T. cruzi* infection

A fraction of 200 µl of the intestinal content was thermally shocked in liquid nitrogen and then treated with digestion solution (10 mM NaCl, 10 mM Tris–HCl, 0.5% SDS, 50 mM EDTA, pH 8.0) and 20 mg/ml of Proteinase K (Invitrogen) at 56 °C for 12–24 h [30]. The DNeasy Blood & Tissue Kit (Qiagen) was applied. Negative extraction controls were included and DNA concentration was estimated using a Quantus™ Fluorometer (Promega).

The detection of BMS was based on the DNA barcoding approach with the application of universal primers to identify vertebrate species, flanking mitochondrial DNA targets of *cyt*b (358 base pairs [bp]) [31] and 12S ribosomal DNA (rDNA; 215 bp) [32], according to the literature. The reconstructive polymerization method to increase DNA quality and concentration was performed as a pre-PCR step [33]. For endogenous control of DNA amplification, a *cox*1 barcode target, which amplified vector gene DNA, was used [34].

Detection of *T. cruzi* infection was based on an 18S rDNA target, using primers and PCR conditions as described in the literature [35]. New primers for an internal region of the *cyt*b target [36], which generate a PCR product of about 200 bp, were designed for this study using Primer3 software 0.4.0 v. Primers MinicytbF: 5′- GCATGAATGTTTTTYAGTTGYT-3′ and MinicytbR: 5′-TCAYACTAAYAAATGYGTRTCAAA-3′ at 20 pmol of each were used in a PCR with a final volume of 25 µl, including, High Fidelity Buffer [600 mM Tris-SO4 (pH 8.9), 180 mM (NH4) 2SO4], 2.5 mM of MgSO_4_, 2 mM of each dNTP, 2.0 U of Platinum^®^ *Taq* DNA Polymerase High Fidelity (Invitrogen, Paisley, Scotland), and 50–100 ng of DNA. PCR thermal conditions were a denaturing step at 94 °C for 2 min followed by 40 cycles of denaturation at 94 °C for 30 s, annealing at 42 °C for 30 s, and elongation at 72 °C for 30 s followed by extension at 72 °C for 7 min in a SimpliAmp Thermal Cycler (Applied Biosystems, CA, USA). Extraction blank and PCR-negative controls were included.

PCR products were subjected to gel electrophoresis agarose (2%) stained with GelRed (Biotium, Inc., CA, USA) and visualized under ultraviolet (UV) light. Amplicons were purified using the Illustra GFX PCR DNA and Gel Band Purification Kit (GE healthcare, IL, USA) or ExoSAP-IT (Affymetrix, USA), according to the manufacturer's instructions. DNA direct sequencing was conducted using the BigDye Terminator v3.1 Cycle Sequencing Kit (Applied Biosystems, CA, USA) and the ABI 3730 sequencer (Applied Biosystems, Waltham, MA, USA) at the RPT01A/FIOCRUZ sequencing facility. The sequences obtained were deposited in GenBank. PCR products from BMS targets were cloned to identify multiple sources as well as different individuals from the same species. *Trypanosoma cruzi* PCR products were cloned when mixed infections were observed in direct DNA sequencing. Molecular cloning was performed using pGEM^®^-T Easy Vector Systems, according to the manufacturer’s protocol (Promega). Cloning products (1–10) were sequenced as described above.

Sequences were submitted to edition, alignment, and visualization using Lasergene SeqMan™ v.7.0 (DNASTAR, Madison, WI, USA), BioEdit v.7.0.5 (Department of Microbiology, North Carolina State University, Raleigh, NC, USA), MUSCLE, and GeneDoc v.2.6.002 [37–39]. BLAST searches were performed in NCBI (http://blast.ncbi.nlm.nih.gov/Blast.cgi) to identify the obtained DNA sequences using the cut-off values of 95% query coverage and 98% genetic identity for species identification. BMS and *T. cruzi* DTU haplotypes from this study and identical sequences from the GenBank reference dataset were recognized using the program DAMBE v.4.0.75 [40]. The intraspecific and intra-DTU genetic distances were calculated using a *p*-distance model in MEGA X v.10.1.7 [41].

Phylogenetic tree construction was performed by applying neighbor-joining (NJ), maximum likelihood (ML), and Bayesian inference (BI) methods. NJ and ML phylogenetic analyses were conducted using MEGA. For the *cyt*b gene marker, NJ method was applied using the Kimura 2-parameter plus gamma distributed rate variation among sites model (K2P + G) and ML, using Hasegawa–Kishino–Yano model plus gamma distribution (HKY + G), as determined by the best-fit model of DNA substitution command using the Bayesian information criterion in MEGA. NJ and ML trees of the 12S rDNA marker were constructed using the K2P and Jukes–Cantor (JC) models, respectively. The statistical support of the branches was generated by 1000 bootstrap reproductions. In addition, BI analysis was subsequently performed (MrBayes), implemented through TOPALi v.2.5 [42], using the best-fit model selected based on the Bayesian information criterion, HKY + G for *cyt*b rDNA and the JC model for 12S rDNA marker.

*Trypanosoma* spp. phylogenetic trees were constructed using NJ and ML, both the K2P + G model for the 18S rDNA target and the Tamura 3-parameter plus gamma distribution (T92 + G) for the *cyt*b marker. BI phylogenetic trees were constructed using K2P + G for 18S rDNA and HKY plus the proportion of invariable sites (HKY + I), for the *cyt*b marker.

Chord diagrams that represent the connections per triatomine between the BMS species/individuals and the *T. cruzi* DTUs, identified in the study, were constructed using the CIRCOS online tool (http://circos.ca/) [43]. The number of clones of BMS and *T. cruzi* DTUs detected per triatomine evaluated was used as a relative metric of abundance [44].

## Results

Twenty-three triatomines were subjected to molecular BMS detection and *Trypanosoma* spp. diagnosis and genotyping analyses. BMS were determined for 21 (91.3%) specimens, 19 using *cyt*b gene target and 10 by 12S rDNA (Table 1). Genetic analyses revealed that the southern tamandua, lesser anteater or southern anteater, *Tamandua tetradactyla* (Linnaeus 1758) (Pilosa, Myrmecophagidae), locally called “tamandua mirim,” was the unique species detected as the BMS of triatomines collected from the South American coati nests, by the two molecular markers applied (Table 1).

**Table 1:** Molecular blood meal source detection and *T. cruzi* genotyping of *T. sordida* specimens collected from a *N. nasua* South American coati nest, Pantanal, Midwest Brazil

Specimens ID	BMS *Tamandua tetradactyla* haplotypes^a^			*Trypanosoma cruzi* infection—DTUs
	*cyt*b	12S rDNA	*cyt*b	18S rDNA
4341	−	−	−	TcII
4342	−	A (1)	−	TcII
4343	A (2)	−	TcI	TcII
4344	A (1)	−	TcI	TcII
4345	A (3)	−	−	−
4346	B (1)	−	−	TcII
4347	B (1), C (1)	A (1)	−	TcII
4348	A (2), B (1)	A (1)	−	TcII
4349	−	−	−	−
4350	A (1)	A (1), B (1)	−	−
4351	A (8), C (1)	A (2)	TcII	TcII
4352	A (3), B (1)	−	TcI	TcII
4353	A (3)	A (1)	TcI	TcII
4354	A (2)	B (1)	TcI	TcII
4355	−	B (1)	−	TcI
4356	A (1)	−	TcI	TcII
4357	A (1)	−	TcI	TcI (2)
4358	A (1)	−	TcI	TcII
4359	A (1)	A (1)	TcI	TcII
4360	A (1)	−	TcI	TcI/TcII
4361	A (1)	−	TcI/TcII	TcI/TcII
4362	A (1)	−	TcI	TcI/TcII
4363	A (1)	A (1)	TcI/TcII	TcI (2)

*BMS* blood meal source ^a^Numbers in parentheses indicate number of clones; “−” indicates negative PCR or cloning

**Fig. 1:**
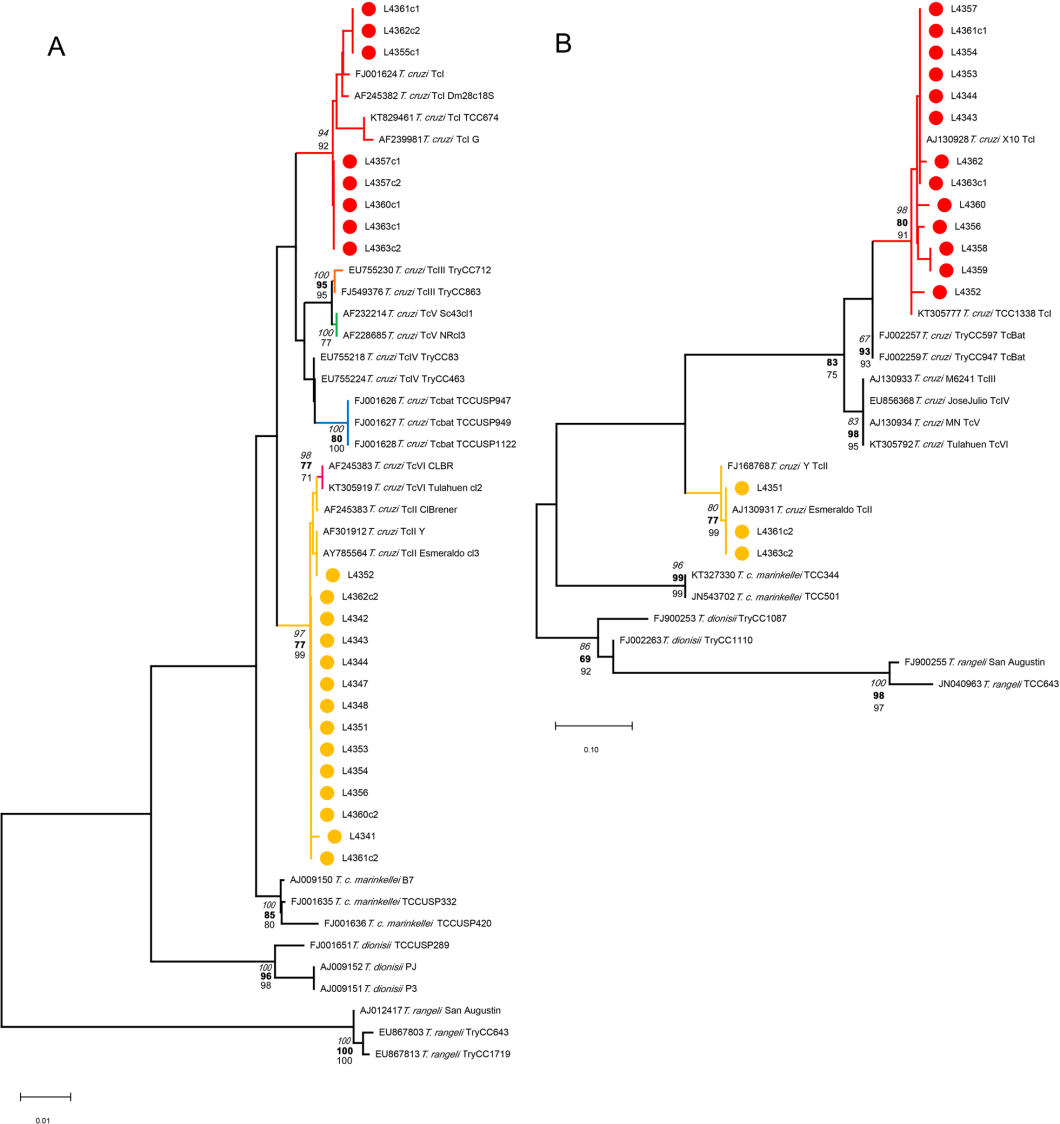
Blood meal source phylogenetic analyses from *T. sordida* specimens collected from a *N. nasua* South American coati nest, Pantanal, Midwest Brazil. **A** *cyt*b gene marker. **B**: 12S rDNA marker. Triangles: samples from this study, colors: haplotypes, red: HapA, yellow: HapB, and green: HapC. Numbers at branches are statistical support based on NJ (bold), ML (regular), and BI (italic) methods. Outgroups: *cyt*b gene, *Euphractus sexcintus* (Linnaeus, 1758) (Cingulata, Dasypodidae)*,* 12S rDNA, *Myrmecophaga tridactyla* (Linnaeus, 1758) (Cingulata, Myrmecophagidae)

The *cyt*b sequences showed a maximum identity of 98.88% (GenBank reference AF232019) and matches up to 97.82% with only *T. tetradactyla* sequences available in GenBank. In the same way, 12S rDNA sequences obtained revealed a maximum identity of 98.62% (GenBank KT818552) and a minimum of 98.14% with only *T. tetradactyla* sequences. Three *cyt*b haplotypes of the southern anteater were identified and two by the 12S rDNA assay, indicating that the bugs fed on a unique *Tamandua* species but from different individuals (Table 1, Fig. 1A, B, Additional file 1: Fig. S1). Considering the BMS *cyt*b marker, 38 clones were obtained from 19 bugs (1–8 clones) grouped in three *T. tetradactyla* haplotypes (HapA–HapC). The most frequent haplotype HapA is characterized by four single-nucleotide polymorphisms (SNPs), A75C, A115G, C353T, and A369T, using the reference sequence GenBank AF232019 (Additional file 1: Fig. S1). The other two haplotypes harbor the same SNPs as HapA, and in addition, HapB harbors C162T, T219A, T225C, C232T, T358C, A396C, and T399C, and HapC harbors A396C (Additional file 1: Fig. S1).

All were synonymous mutations that did not alter the encoded amino acid, with no effect on the resulting mitochondrial protein.

The *cyt*b dataset was constructed using all *T. tetradactyla* sequences available (*n* = 111) in GenBank (05/2022), without short identical and short sequences, resulting in a final dataset of 215 bp (*n* = 9). The phylogenetic tree showed that HapA and HapC seem to be related, despite the clear A396C SNP in the alignment (see Additional file 1: Fig. S1). However, the *T. tetradactyla* HapB was visibly quite distant from the other two individuals (Fig. 1A). The genetic *p*-distance between *cyt*b haplotypes HapA-HapB and HapC-HapB was the same, 0.0203 (± standard error, SE = 0.0074) in contrast to the close related HapA-HapC of 0.0029 (SE = 0.0029). The mean genetic distance is 0.0145 (SE = 0.0057).

Most of the bugs, 16/19, fed on anteater HapA, four bugs on anteater HapB, and two bugs on anteater HapC (Table 1, Fig. 1A, C). Regarding single and multiple BMS per bug specimen, a total of 15 *T. sordida* insects fed on a single animal (14 bugs on anteater HapA and one on anteater HapB), and four bugs fed on two different *T. tetradactyla* animals (two on HapA+C, one HapA+B, and HapA+C) (Table 1).

The 12S rDNA assay was less effective, with 12 clones of *T. tetradactyla* as BMS in 10 bugs, but with results in two bugs previously negative by *cyt*b marker (Table 1). Two different anteater animals were detected (Fig. 1B) presenting the following SNPs: HapA: A659G, C696T, T697C, and HapB: C696T, T697C, using KT818552 as a reference (see Additional file 2, Fig. S2). The genetic *p*-distance between haplotypes HapA and HapB was 0.0046 (SE = 0.0047). Most of the bugs fed on a single anteater, seven on animal HapA, two on animal HapB, and one bug on two anteaters HapA+B. Combining all BMS genetic data, it is possible to establish that *cyt*b HapA and 12S rDNA HapA correspond to the same anteater animal (exception, specimen 4347, fed on *cyt*b HapB+HapC and 12S rDNA HapA). However, it is not possible to see a parallel with 12S rDNA HapB (Table 1). The integration of both molecular markers results in the possibility of more bugs with a multiple feeding profile, including three different anteater individuals serving as BMS of the same *T. sordida* specimen (Table 1).

**Fig. 2:**
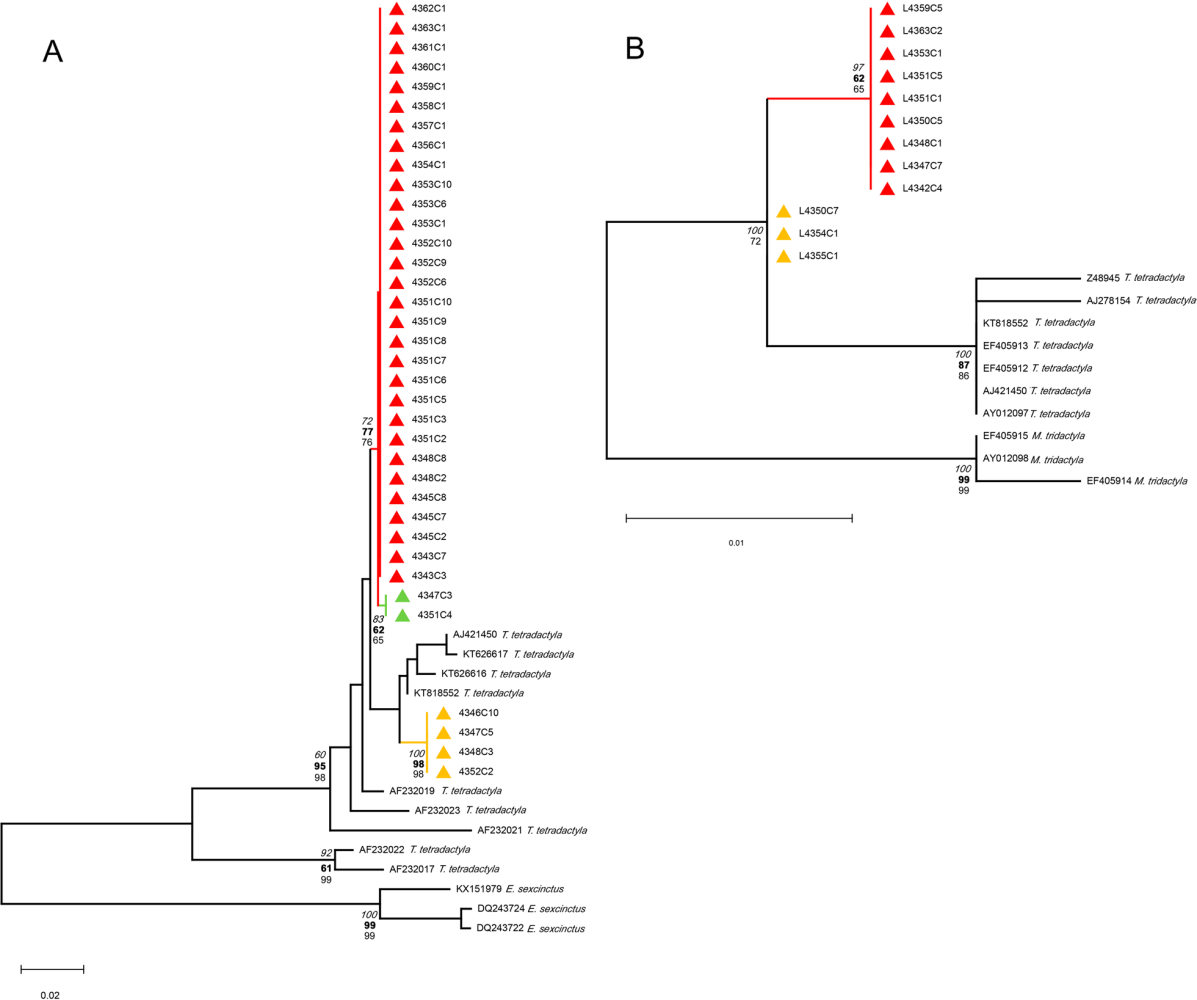
*Trypanosoma* spp. phylogenetic analyses from *T. sordida* specimens collected from a *N. nasua* South American coati nest, Pantanal, Midwest Brazil. **A** 18S rDNA marker. **B** *cyt*b gene marker. Circles: Samples from this study, red: TcI and yellow: TcII. Reference sequences indicating *Trypanosoma* sp. or *T. cruzi* DTUs and GenBank accession numbers. Numbers at branches are statistical support based on NJ (bold), ML (regular), and BI (italic) methods

Regarding *T. cruzi* infection, 86.95% (20/23) of *T. sordida* bugs were positive, based on two genetic markers, six specimens by DTU TcII, two by TcI, and 12 in mixed infection by these lineages (Table 1). 18S rDNA TcII sequences were obtained from 17 insects and TcI from eight (Fig. 2A). Two different 18S rDNA haplotypes were observed in each DTU. One TcII haplotype was identical to strain Y (AF301912) and Esmeraldo (AY785564) reference sequences, and the second one, from specimen 4341, with only an SNP C860T, using the same reference sequences (inter-haplotypes *p*-distance = 0.0015). The TcI haplotype from specimens 4355c1, 4361c1, and 4362c1 grouped together in a sub-cluster (Fig. 2A) and had a sequence with two SNPs, del1048T and C1049T, using Dm28c strain sequence as reference (AF245382). The remaining specimens harbored a TcI haplotype with the same two SNPs observed, plus A1137G and a six-bp gap at the 1138 position (AF245382) (inter-haplotypes *P*-distance = 0.0015). The application of the *cyt*b target detected 13 insects with TcI, and three with TcII genotype, showing a haplotype sequence identical to the Esmeraldo strain (AJ130931) (Fig. 2B). On the other hand, a high intra-DTU TcI diversity was revealed with six different *cyt*b haplotypes—an individual haplotype in each specimen 4352, 4356, 4360, and 4362, the same haplotype observed in both 4358 and 4359 bugs—and the remaining specimens had a haplotype sequence identical to the Sylvio X10 strain (AJ130928) (Fig. 2B). The TcII haplotype pairwise distance ranged from 0.0072 to 0.0216, with a mean genetic distance of 0.0117 (SE = 0.0042). The 18S rDNA phylogeny defined species-specific and DTU-specific clades with the exception of TcII/VI and TcIII/TcV clusters (Fig. 2A). However, the discrimination between TcII and TcVI in the *cyt*b phylogeny was clearly observed, with moderate support (Fig. 2B), solving the DTU TcII genotyping.

**Fig. 3:**
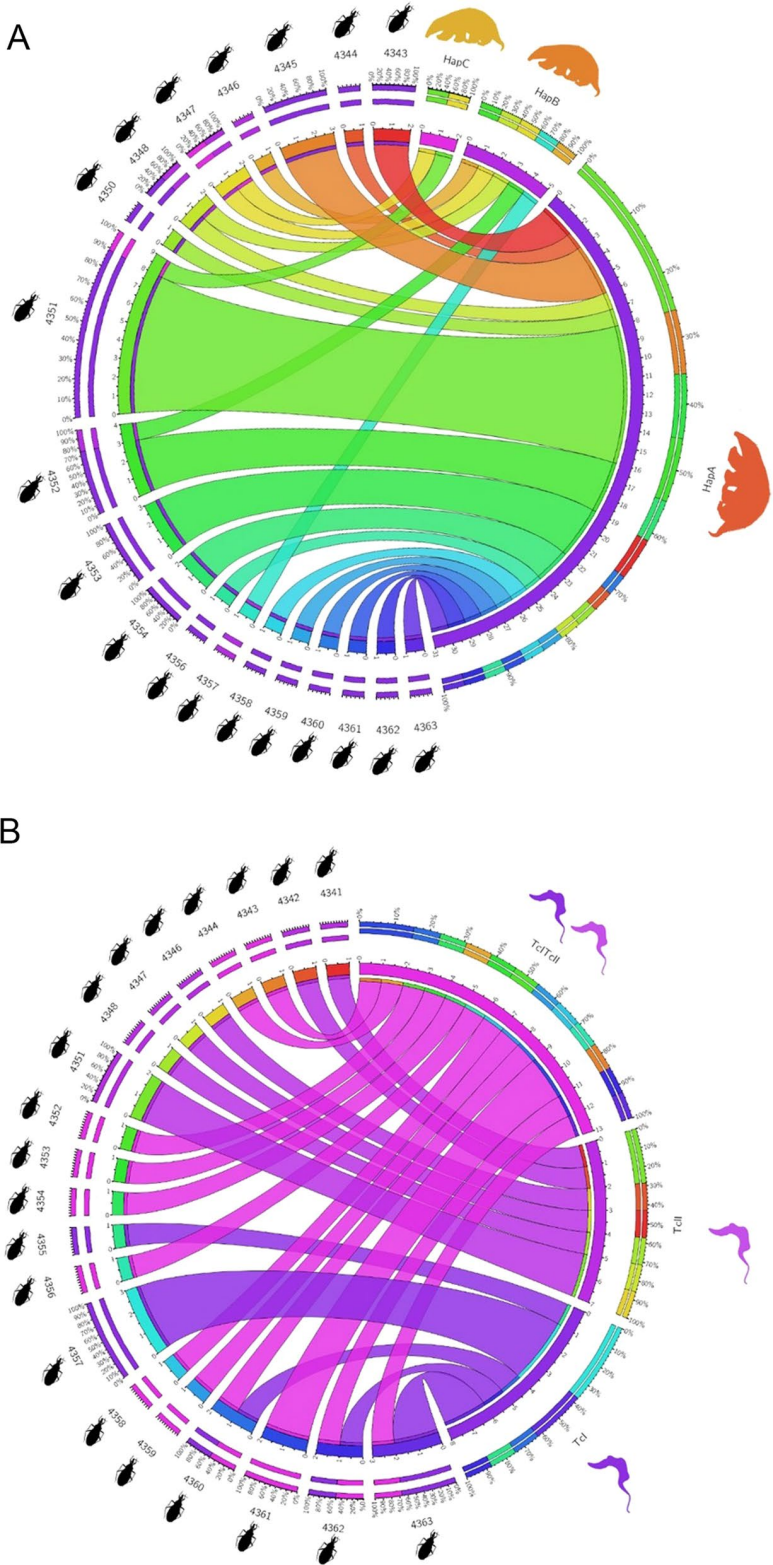
Chord circular diagrams representing networks of the relative abundance of blood meal sources and *T. cruzi* DTUs detected per *T. sordida* specimen from this study collected from *N. nasua* South American coati nest, Pantanal, Midwest Brazil. **A** BMS *T. tetradactyla* haplotypes/individuals Hap A–C based on the *cyt*b gene. **B** *T. cruzi* DTUs TcI, TcII, and mixed-infection TcI/TcII

The chord circular network of *cyt*b BMS clearly demonstrated that most of the insects fed mainly on the individual HapA from the three *T. tetradactyla* animals identified (Fig. 3A). However, individuals of HapB and HapC served concomitantly as BMS but with less frequency. The chord circular diagram of *T. cruzi* genotyping showed the predominance of TcI/TcII mixed infection, computing the DTUs detected per *T. sordida* specimen from this study (Fig. 3B). No evident difference in the proportion of insects positive exclusively to TcI versus TcII was observed.

In an attempt to integrate BMS and *T. cruzi* genotyping data from triatomines, most of the bugs that feed on anteater HapA were *T. cruzi*-infected triatomines TcI/TcII, two were TcI positive and two negative. The triatomines feed on animal HapB, less frequent and unrelated to anteater HapA, were mostly infected by a single TcII and one insect with mixed infection. In the anteater animal HapC, closely related to HapA, triatomines were always fed on A+B anteaters and were only TcII-positive.

## Discussion

*Trypanosoma cruzi* infection by single and mixed DTUs occurs in several wild mammals and reduviid species in almost all natural environments in the Neotropical realm [3]. Since most outbreaks of Chagas disease in humans originate in the wild ecotope, it is essential to understand the ecology of wild *T. cruzi* lineages, as well as the role of host species involved in the natural transmission cycles. In this sense, the discrimination of the BMS of triatomines provides data about the host species of the faunal community in the enzootic areas. The genetic detection of BMS is still little explored in Brazil, despite being a highly specific and sensitive methodology as well as a strategic instrument for understanding the ecology, at different levels, of American trypanosomiasis and other vector-borne infectious diseases. This is the first report of genetic BMS of triatomines in the Pantanal biome and the first in the country to use molecular cloning to achieve multi-host diversity per hematophagous vectors.

A unique molecular BMS study performed on *T. sordida* used four genetic markers for humans, dogs, cats, and birds, yielding generic results, and no other vertebrate could be detected as BMS [20]. *Triatoma sordida* was historically indicated as the unique species of the genus with a BMS preference for birds [45]. However, there are some BMS reports of other vertebrates, such as a variety of wild mammals including rodents, bats, and marsupials, a number of domestic animals, and even reptiles, detected by precipitin tests [46]. Together with our finding that *T. sordida* can also feed on southern anteaters, the eclectic behavior of this triatomine species, rather than BMS preference, accepting a range of alternative sources is confirmed. As noted by Rabinovich et al. [47], the proximity and availability of a sort of host vertebrates may influence the opportunistic character of some triatomines.

Olifiers et al. [13] reported that South American coatis built arboreal nests with branches, leaves, and vines intertwined that were used for rest and reproduction. Additionally, de Lima et al. [9] showed that coatis’ nests can also be explored by other animals such as marsupials, rodents, and birds, based on precipitin BMS tests. These observations may explain the maintenance of triatomine colonies, including the non-volant nymphs, in nests abandoned by South American coatis [9]. It was also observed that the coati nests may be visited by the spiny rat, *Thrichomys fosteri*, valid name *T. pachyurus* (Wagner, 1845) (Rodentia, Echimyidae), a scansorial species that has been reported with high frequency and parasitemia by *T. cruzi* (TcI) [4, 5, 15]. This caviomorph rodent species was also recorded inside South American coatis’ nests using camera traps to monitor it [3]. In the present study, the results of genetic BMS identification of triatomines collected from a South American coati’s nest showed that these reduviid bugs fed on *T. tetradactyla* individuals, revealing that this habitat can also be occupied by this Pilosa species.

Given these shared occupations and the presence of infected triatomines, coatis’ nests can be considered an important core for maintaining different genotypes of *T. cruzi*. In fact, different DTUs including TcI, TcII, TcIII, and TcIV have been observed in hosts from the Pantanal biome, with the highest frequencies with both TcI and TcII, mainly in mixed infection [3, 16, 48]. In addition, *Nasua nasua* was indicated as the mammal responsible for retaining high parasitemia by both TcI and TcII in the biome, and mixed infections by different *T. cruzi* lineages have been recorded in the top predator species [17]. Herein, TcI and TcII are confirmed in *T. sordida* that inhabit a South American coati nest, with a high frequency of TcI/TcII mixed infection and intra-DTU diversity, especially from TcI. The detection of haplotypes of classic reference strains (Sylvio X10, Y, and Esmeraldo) in the triatomine studied in this work showed that they are still circulating in the wild, together with other DTU variants. The heterogeneity of *T. cruzi* TcI was previously observed in wild mammals from diverse Brazilian biomes [49, 50], including from the Pantanal biome [49, 51], and in triatomine vectors [51], using high-resolution approaches, multilocus sequence typing (MLST) and multilocus microsatellite typing (MLMT). In this study, the mixed DTU infections per vector were detected due to the use of two molecular markers and the discriminatory capacity of the Barcoding DNA and molecular cloning methodologies. We highlighted that the *cyt*b primers designed for this study were useful in complementing the *T. cruzi* diagnosis and genotyping based on 18S rDNA, and the target region was enough polymorphic and informative despite the small amplicon size generated, which also allowed some intra-DTU resolution, at least to TcI and TcII. We recommend the parasite diagnosis based on these two molecular markers, plus cloning, which is not frequently applied. It is possible that MLST and MLMT markers could reveal higher intra-TcI diversity in the triatomines examined, due to their remarkable resolution power. Therefore, mixed DTU and haplotype profiles must be more recurrent in this Pantanal subregion than those detected by conventional techniques, with consequences for the intricate *T. cruzi* transmission networks and for the fitness of the mammal hosts, which is still unknown.

The results of the present study suggest that TcI and TcII infection in the region may not be driven only by coatis, but apparently, southern tamandua, who also present high parasitemia in the studied area [12], seems to have a role when multiple DTU infections, and certainly reinfections, occur mediated by vectors during the shared coati nest occupation. Aside from southern anteaters being poorly studied, we are aware that the results of the present research should be corroborated by sampling more coati nests and in other subregions of the Pantanal biome. It is important to mention that nest colonization with triatomines is highly variable, with rates from 33.3% to 9.6%, even in the same landscape [9, 12]. This could be related to specific ecological characteristics of areas [12], and undoubtedly, to the BMS availability in the nests. However, the rate of *T. cruzi* infection of triatomines collected from coati nests can reach 70% per single nest [12].

*Tamandua tetradactyla* is an autochthonous South American mammal species, occurring in all Brazilian biomes [52]. It is primarily arboreal, using branches for moving and foraging, but can also move, feed, and rest on the ground. It has a predominantly nocturnal habit, and trees, burrows of armadillos, or other natural cavities are indicated as the preferable spaces to rest [53]. *Tamandua tetradactyla* feeds on insects, mostly nests of ants or termites, from the ground or in trees [53].

A *T. cruzi* infection in the southern tamandua has already been reported. In 1942, the first report in the Amazon basin was on three animals by parasitological direct tests of fresh blood and xenodiagnosis [54]. Also, in the Amazon rainforest, de Araújoet al. [55] revealed mixed infection by *Trypanosoma rangeli*, *Leishmania infantum*, and *T. cruzi* TcI, using mini-exon gene analysis. Recently, Santos et al. [5] reported for the first time *T. cruzi* infection in *T. tetradactyla* from the Pantanal wetland, the site of the present study. The TcI lineage was isolated from hemocultures, indicating high parasitemias. In the present work, anteaters are the unique BMS from *T. sordida* specimens infected by TcI, TcII, and mixed TcI/TcII.

Regarding the BMS methodology, it was possible to detect three different *T. tetradactyla* haplotypes using the *cyt*b marker and two haplotypes based on 12S rDNA. Since the *cyt*b marker is the most variable target [22], it was expected that more *cyt*b haplotypes would be identified than the 12S rDNA target. Furthermore, the vertebrate *cyt*b sequence dataset available in GenBank is richer than the 12S rDNA dataset. It is possible that the two closely related *cyt*b haplotypes HapA and HapC harbor the same 12S rDNA haplotype. The limited genetic variability of 12S rDNA and the lack of taxonomic definition in the discrimination of some species of Caprinae and Didelphinae were observed [24]. However, due to the small amplicon size, it is efficient in revealing BMS in degraded DNA [22], and as demonstrated in the present study, in previously negative samples by using the *cyt*b marker (Table 1). The pros and cons of these molecular markers make the use of both in parallel the best methodological strategy in the detection and identification of vertebrate species acting as BMS.

Three southern tamandua individuals were the BMS of *T. sordida* collected from the coati nest. Medri et al. [52] reported that the parental care of a single offspring usually lasts 1 year; it is carried on the mother's back or left in “a nest” when it is going to feed. Since anteaters have an individualist nonsocial behavior, the three individuals identified in the present study occupied the nest at different times. Another possibility is that two individuals corresponding to a mother and her single offspring, potentially the closely related animals HapA and HapC, and the other individual, possibly the genetically distant anteater HapB, visited the nest at different times. However, as maternally related animals may have the same mitochondrial DNA (mtDNA) haplotype, it is plausible that more than three different individuals of *T. tetradactyla* visited the coati nest.

We could also hypothesize that the massive frequency of animal HapA could represent a long-lasting exploration of the coati nest, while the other animals could be punctual events or old previous visits, reflected by low BMS DNA detection. However, an initial massive feeding by fasting triatomines in anteater individual HapA, and after their feeding on individuals HapB and Hap C, could not be ruled out.

In this regard, the absence of coati DNA detection as BMS of the bugs collected from their nest suggests the persisting exploration of anteaters in the coati nest, and the total abandonment by the “constructors.” Abandoned coati nests have been reported in the literature [4, 9, 13]. Recently, it was demonstrated that BMS can be detected in triatomines until 12 weeks after the last feeding, using the same molecular method applied in this study, under experimental conditions [55]. Proportionally speaking, we are inclined to suggest that the coatis abandoned this nest for months, which was later used only by anteaters. The direction of the transmission, from triatomines to anteaters or vice versa, is not possible to determine, but the coati nest acting as a hub of the *T. cruzi* transmission is clear.

The analysis of BMS from the triatomine vector is a relevant and non-faunal invasive approach for gathering information on which wild mammals participate in a local transmission network. The data presented herein implicate the *T. tetradactyla* participation in the *T. cruzi* enzootic scenario in this Pantanal subregion, not only for this niche occupation but also due to its ecological peculiarities in exploring the arboreal and terrestrial strata.

Desbiez and Kluyber (2013) reported that *T. tetradactyla* was the most frequent vertebrate using burrows of giant armadillo, “the ecosystem engineers,” from other 24 species documented in the Brazilian Pantanal [56]. *Tamandua tetradactyla* was found as the only feeding source by molecular BMS analysis in 14/31 *Rhodnius robustus* collected in an *Attalea phalerata* palm tree crown from the Brazilian Amazon region, and was suggested as an important reservoir for *T. rangeli* [57]. Since *T. tetradactyla* has been found in arboreal coati nests, giant armadillo burrows, and palm tree crows, this anteater species may be connecting different *T. cruzi* transmission cycles that would be occurring in the canopy and on the ground.

As bioaccumulator of *T. cruzi* DTUs, South American coatis were proposed as a transmission hub linking different sylvatic cycles [15, 17]. However, would the South American coatis themselves or what they build be considered as hubs of *T. cruzi* cycle transmission? In the strict sense, it would not be them, but their nests. South American coatis build the structure and modify the environment, so they are responsible for their origin, but even within the species, there is no exclusivity of use for the animals that build them. Indeed, based on our results and previous data on serological tests and camera traps [3, 9], there is no use restricted to the South American coati species. As highlighted earlier, arboreal nests represent an example of the richness of possibilities of encounters between mammals and reduviid species for *T. cruzi* transmission [9]. Therefore, these nests have intra- and interspecific communal use for those who explore the arboreal stratum and consequently act as *T. cruzi* transmission hubs, available to all the competent hosts that potentially could frequent them.

## Conclusion

The genetic identification approach of multiple BMS used in this study provided a powerful methodology for reaching the taxonomic level of species and different individuals of the same host species. The study reveals non-obviously different species and ecologically relevant information on faunal diversity implicated in parasite transmission in the wild. The methodology also allowed us to identify a predominance of *T. cruzi* mixed infections, with relevant and still unknown consequences for parasite transmission networks and for mammal fitness. Southern anteaters can occupy the South American coatis’ nests, serving as BMS of *T. sordida* specimens. We propose that the coatis’ nests are the true hubs of the ecological *T. cruzi* transmission in Pantanal, and not the coatis themselves, as previously suggested. This approach is also recommended for application to the study of the ecology and epidemiology of other vector-borne diseases.

## Supplementary Information

**Additional file 1**: **Figure S1** The sequence alignments of the *cyt*b marker for BMS detection of *T. sordida* from a *N. nasua* South American coati nest, Pantanal, Midwest Brazil.

**Additional file 2**: **Figure S2** The sequence alignments of the 12S rDNA marker for BMS detection of *T. sordida* from a *N. nasua* South American coati nest, Pantanal, Midwest Brazil.

## Data Availability

All data generated or analyzed during this study are included in this article and its additional files.

## Abbreviations

BMS: Blood meal source
DTU: Discrete typing unit
