# Supplementary material for: “Mi Casa, Tu Casa”: the coati nest as a hub of Trypanosoma cruzi transmission in the southern Pantanal biome revealed by molecular blood meal source identification in triatomines
Source: Parasit Vectors. 2023 Jan 23;16:26. doi: 10.1186/s13071-022-05616-w (PMC9872340; doi:10.1186/s13071-022-05616-w)
Supplement: Supplementary file 1 — Additional file 1: Figure S1 The sequence alignments of the cytb marker for BMS detection of T. sordida from a N. nasua South American coati nest, Pantanal, Midwest Brazil. [file 13071_2022_5616_MOESM1_ESM.pdf]

```
                       70        80        90        100       110       120       130       140       150       160
                       |....|....|....|....|....|....|....|....|....|....|....|....|....|....|....|....|....|....|....|....
AF232019 Ttetradactyla CCATCAAACATCTCAGCATGATGAAACTTCGGATCCCTACTAGGAATCTGCCTAGTAATCCAAATCCTAACAGGCCTCTTTTTAGCAATACACTATACAT
KT818552 Ttetradactyla .............................................G..............................................T.......
KT626616 Ttetradactyla .............................................G..................C...........................T..C....
KT818551 Tmexicana     .............................................G.A............................................T..C....
AJ421450 Ttetradactyla ...........T.................................G..................C...........................T.......
AF232023 Ttetradactyla ................................G............G............................................T.T.......
KT626617 Ttetradactyla ...........T.................................G..................C...........................T.......
AF232021 Ttetradactyla ...........T...T................G.....C..G.C..........C..C.G.....T...........A..CC....C........C...C
AF232022 Ttetradactyla ........T..T..................A....T...........T.....GA.T....................A..............T..C....
AF232017 Ttetradactyla ........T..T.......................T...........T.....GA.T....................A..............T..C....
4343C3_cytFA           .....C.......................................G......................................................
4343C7_cytFA           .....C.......................................G......................................................
4345C2_cytFA           .....C.......................................G......................................................
4345C7_cytFA           .....C.......................................G......................................................
4346C10_cytFA          .....C.......................................G..............................................T.......
4347C5_cytFA           .....C.......................................G..............................................T.......
4348C3_cytFA           .....C.......................................G..............................................T.......
4352C2_cytFA           .....C.......................................G..............................................T.......
4347C3_cytFA           .....C.......................................G......................................................
4351C4_cytFA           .....C.......................................G......................................................
4345C8_cytFA           .....C.......................................G......................................................
4348C2_cytFA           .....C.......................................G......................................................
4348C8_cytFA           .....C.......................................G......................................................
4351C2_cytFA           .....C.......................................G......................................................
4351C3_cytFA           .....C.......................................G......................................................
4351C5_cytFA           .....C.......................................G......................................................
4351C6_cytFA           .....C.......................................G......................................................
4351C7_cytFA           .....C.......................................G......................................................
4351C8_cytFA           .....C.......................................G......................................................
4351C9_cytFA           .....C.......................................G......................................................
4351C10_cytFA          .....C.......................................G......................................................
4352C6_cytFA           .....C.......................................G......................................................
4352C9_cytFA           .....C.......................................G......................................................
4352C10_cytFA          .....C.......................................G......................................................
4353C1_cytFA           .....C.......................................G......................................................
4353C6_cytFA           .....C.......................................G......................................................
4353C10_cytFA          .....C.......................................G......................................................
4354C1_cytFA           .....C.......................................G......................................................
4356C1_cytFA           .....C.......................................G......................................................
4357C1_cytFA           .....C.......................................G......................................................
4358C1_cytFA           .....C.......................................G......................................................
4359C1_cytFA           .....C.......................................G......................................................
4360C1_cytFA           .....C.......................................G......................................................
4361C1_cytFA           .....C.......................................G......................................................
4362C1_cytFA           .....C.......................................G......................................................
4363C1_cytFA           .....C.......................................G......................................................
```

```
                         170       180       190       200       210       220       230       240       250       260
                         |....|....|....|....|....|....|....|....|....|....|....|....|....|....|....|....|....|....|....|....
AF232019 Ttetradactyla   CAGACACAATCACAGCATTTTCATCAGTCACCCATATCTGCCGAGATGTTAATTATGGATGACTAATTCGCTATATCCATGCAAACGGGGCCTCAATATT
KT818552 Ttetradactyla   .......................................................C......T.....................................
KT626616 Ttetradactyla   .......................................................C......T.....................................
KT818551 Tmexicana       .......................................................C............................................
AJ421450 Ttetradactyla   ...................C...................................C......T.....................................
AF232023 Ttetradactyla   ............................N..........................C...........C................................
KT626617 Ttetradactyla   ...................C...................................C......T.....................................
AF232021 Ttetradactyla   .........C..........................................................................................
AF232022 Ttetradactyla   ...................C................................C..............G.....C.....C..C.................
AF232017 Ttetradactyla   ...................C................................C..............C.....C.....C..C.G...............
4343C3_cytFA             ....................................................................................................
4343C7_cytFA             ....................................................................................................
4345C2_cytFA             ....................................................................................................
4345C7_cytFA             ....................................................................................................
4346C10_cytFA            .................................................A.....C......T.....................................
4347C5_cytFA             .................................................A.....C......T.....................................
4348C3_cytFA             .................................................A.....C......T.....................................
4352C2_cytFA             .................................................A.....C......T.....................................
4347C3_cytFA             ....................................................................................................
4351C4_cytFA             ....................................................................................................
4345C8_cytFA             ....................................................................................................
4348C2_cytFA             ....................................................................................................
4348C8_cytFA             ....................................................................................................
4351C2_cytFA             ....................................................................................................
4351C3_cytFA             ....................................................................................................
4351C5_cytFA             ....................................................................................................
4351C6_cytFA             ....................................................................................................
4351C7_cytFA             ....................................................................................................
4351C8_cytFA             ....................................................................................................
4351C9_cytFA             ....................................................................................................
4351C10_cytFA            ....................................................................................................
4352C6_cytFA             ....................................................................................................
4352C9_cytFA             ....................................................................................................
4352C10_cytFA            ....................................................................................................
4353C1_cytFA             ....................................................................................................
4353C6_cytFA             ....................................................................................................
4353C10_cytFA            ....................................................................................................
4354C1_cytFA             ....................................................................................................
4356C1_cytFA             ....................................................................................................
4357C1_cytFA             ....................................................................................................
4358C1_cytFA             ....................................................................................................
4359C1_cytFA             ....................................................................................................
4360C1_cytFA             ....................................................................................................
4361C1_cytFA             ....................................................................................................
4362C1_cytFA             ....................................................................................................
4363C1_cytFA             ....................................................................................................
```

```
                           270       280       290       300       310       320       330       340       350       360
                           |....|....|....|....|....|....|....|....|....|....|....|....|....|....|....|....|....|....|....|....
AF232019 Ttetradactyla     CTTCATATGCCTATACCTACATGTAGGTCGTGGCCTATACTATGGCTCCTACTTATACCTAGAAACTTGAAACATCGGAGTAACCCTCTTATTAGCCACA
KT818552 Ttetradactyla     ...................................................................................T...............T
KT626616 Ttetradactyla     ...................................................................................T...............T
KT818551 Tmexicana         ......................A....................................................T.......T.......C.......T
AJ421450 Ttetradactyla     ...................................................................................T.......C.......T
AF232023 Ttetradactyla     ....................................G...............C..............................T...............T
KT626617 Ttetradactyla     ..........................................C........................................T.......C.......T
AF232021 Ttetradactyla     ..............................................................................N....T...............T
AF232022 Ttetradactyla     ...T.......................C..C.....G........G......C.....T.............T..........TT...............
AF232017 Ttetradactyla     ...T.......................C..C.....G........G...C..C.....T.............T..T.......TT...............
4343C3_cytFA               ...................................................................................T...............T
4343C7_cytFA               ...................................................................................T...............T
4345C2_cytFA               ...................................................................................T...............T
4345C7_cytFA               ...................................................................................T...............T
4346C10_cytFA              ...................................................................................T....C..........T
4347C5_cytFA               ...................................................................................T....C..........T
4348C3_cytFA               ...................................................................................T....C..........T
4352C2_cytFA               ...................................................................................T....C..........T
4347C3_cytFA               ...................................................................................T...............T
4351C4_cytFA               ...................................................................................T...............T
4345C8_cytFA               ...................................................................................T...............T
4348C2_cytFA               ...................................................................................T...............T
4348C8_cytFA               ...................................................................................T...............T
4351C2_cytFA               ...................................................................................T...............T
4351C3_cytFA               ...................................................................................T...............T
4351C5_cytFA               ...................................................................................T...............T
4351C6_cytFA               ...................................................................................T...............T
4351C7_cytFA               ...................................................................................T...............T
4351C8_cytFA               ...................................................................................T...............T
4351C9_cytFA               ...................................................................................T...............T
4351C10_cytFA              ...................................................................................T...............T
4352C6_cytFA               ...................................................................................T...............T
4352C9_cytFA               ...................................................................................T...............T
4352C10_cytFA              ...................................................................................T...............T
4353C1_cytFA               ...................................................................................T...............T
4353C6_cytFA               ...................................................................................T...............T
4353C10_cytFA              ...................................................................................T...............T
4354C1_cytFA               ...................................................................................T...............T
4356C1_cytFA               ...................................................................................T...............T
4357C1_cytFA               ...................................................................................T...............T
4358C1_cytFA               ...................................................................................T...............T
4359C1_cytFA               ...................................................................................T...............T
4360C1_cytFA               ...................................................................................T...............T
4361C1_cytFA               ...................................................................................T...............T
4362C1_cytFA               ...................................................................................T...............T
4363C1_cytFA               ...................................................................................T...............T
```

```
                          370       380       390       400       410       420
                          |....|....|....|....|....|....|....|....|....|....|....|
AF232019 Ttetradactyla    ATAGCAACCGCATTCATGGGCTATGTACTTCCATGAGGACAAATATCATTCTGAGG
KT818552 Ttetradactyla    .............................C..........................
KT626616 Ttetradactyla    ..........................G..C..........................
KT818551 Tmexicana        .......................C................................
AJ421450 Ttetradactyla    .............................C..........................
AF232023 Ttetradactyla    .......................C................................
KT626617 Ttetradactyla    .............................C..........................
AF232021 Ttetradactyla    ........................................................
AF232022 Ttetradactyla    ........A...........A..C..C.............................
AF232017 Ttetradactyla    .........A..........A..C..C.............................
4343C3_cytFA              ........................................................
4343C7_cytFA              ........................................................
4345C2_cytFA              ........................................................
4345C7_cytFA              .....................................................
4346C10_cytFA             ..........................C..C..........................
4347C5_cytFA              ..........................C..C..........................
4348C3_cytFA              ..........................C..C..........................
4352C2_cytFA              ..........................C..C..........................
4347C3_cytFA              ..........................G.............................
4351C4_cytFA              ..........................G.............................
4345C8_cytFA              ........................................................
4348C2_cytFA              ........................................................
4348C8_cytFA              ........................................................
4351C2_cytFA              ........................................................
4351C3_cytFA              ........................................................
4351C5_cytFA              ........................................................
4351C6_cytFA              ........................................................
4351C7_cytFA              ........................................................
4351C8_cytFA              ........................................................
4351C9_cytFA              ........................................................
4351C10_cytFA             ........................................................
4352C6_cytFA              ........................................................
4352C9_cytFA              ........................................................
4352C10_cytFA             ........................................................
4353C1_cytFA              ........................................................
4353C6_cytFA              ........................................................
4353C10_cytFA             ........................................................
4354C1_cytFA              ........................................................
4356C1_cytFA              ........................................................
4357C1_cytFA              ........................................................
4358C1_cytFA              ........................................................
4359C1_cytFA              ........................................................
4360C1_cytFA              ........................................................
4361C1_cytFA              ........................................................
4362C1_cytFA              ........................................................
4363C1_cytFA              ........................................................
```
