# Supplementary material for: “Mi Casa, Tu Casa”: the coati nest as a hub of Trypanosoma cruzi transmission in the southern Pantanal biome revealed by molecular blood meal source identification in triatomines
Source: Parasit Vectors. 2023 Jan 23;16:26. doi: 10.1186/s13071-022-05616-w (PMC9872340; doi:10.1186/s13071-022-05616-w)
Supplement: Supplementary file 2 — Additional file 2: Figure S2 The sequence alignments of the 12S rDNA marker for BMS detection of T. sordida from a N. nasua South American coati nest, Pantanal, Midwest Brazil. [file 13071_2022_5616_MOESM2_ESM.pdf]

```
                            500       510       520       530       540       550       560       570       580       590       600
                            ..|....|....|....|....|....|....|....|....|....|....|....|....|....|....|....|....|....|....|....|....|....|..
KT818552 T.tetradactyla     CAAACTGGGATTAGATACCCCACTATGCCTAGCCGTAAACACAAATATTCCAACAACAAGAATATTCGCCAGAGTACTACTAGCAACAGCTAAAAACTCAAAGGACTTGG
EF405913 T.tetradactyla     ..............................................................................................................
EF405912 T.tetradactyla     ..............................................................................................................
AJ421450 T.tetradactyla     ..............................................................................................................
AY012097 T.tetradactyla     ..............................................................................................................
KT818551 T.mexicana         ................................T.............................................................................
Z48945  T.tetradactyla      ................................T.............................................................................
AJ278154 T.tetradactyla     ............................................................................................G.................
L4342C4                     ..............................................................................................................
L4347C7                     ..............................................................................................................
L4348C1                     ..............................................................................................................
L4350C5                     ..............................................................................................................
L4351C1                     ..............................................................................................................
L4351C5                     ..............................................................................................................
L4353C1                     ..............................................................................................................
L4359C5                     ..............................................................................................................
L4363C2                     ..............................................................................................................
L4350C7                     ..............................................................................................................
L4354C1                     ..............................................................................................................
L4355C1                     ..............................................................................................................
EF405915 M.tridactyla       .........................................T........A...................................T...........T...........
AY012098 M.tridactyla       .........................................T........A...................................T...........T...........
EF405914 M.tridactyla       .........................................T........A...................................T...........T...........

                              610       620       630       640       650       660       670       680       690       700       710
                            ..|....|....|....|....|....|....|....|....|....|....|....|....|....|....|....|....|....|....|....|....|..
KT818552 T.tetradactyla     CGGTGCTTCATATCCACCTAGAGGAGCCTGTTCTATAATCGATAAACCCCGATAAACCTCACCACCTCTTGCTAATACAGCCTATATACCGCCATCTCTAGCAAA
EF405913 T.tetradactyla     .........................................................................................................
EF405912 T.tetradactyla     .........................................................................................................
AJ421450 T.tetradactyla     .........................................................................................................
AY012097 T.tetradactyla     .........................................................................................................
KT818551 T.mexicana         .........................................................................................................
Z48945  T.tetradactyla      .........................................................................................................
AJ278154 T.tetradactyla     .........................................................................................................
L4342C4                     ............................................................G....................................TC......
L4347C7                     ............................................................G....................................TC......
L4348C1                     ............................................................G....................................TC......
L4350C5                     ............................................................G....................................TC......
L4351C1                     ............................................................G....................................TC......
L4351C5                     ............................................................G....................................TC......
L4353C1                     ............................................................G....................................TC......
L4359C5                     ............................................................G....................................TC......
L4363C2                     ............................................................G....................................TC......
L4350C7                     .................................................................................................TC......
L4354C1                     .................................................................................................TC......
L4355C1                     .................................................................................................TC......
EF405915 M.tridactyla       ............................................................................G....................TC......
AY012098 M.tridactyla       ............................................................................G....................TC......
EF405914 M.tridactyla       .................................................................T..........G....................TC......
```
